# Imaging Features and Clinical Outcomes of Ischemic Cholangiopathy After Drug-Eluting Bead Transarterial Chemoembolization for Hepatocellular Carcinoma: A Retrospective Single-Center Cohort Study and Literature Review

**DOI:** 10.3390/curroncol33050254

**Published:** 2026-04-28

**Authors:** Saša Štupar, Peter Popović, Rok Dežman, Jure Uršič, Martin Zaplotnik, Miha Štabuc, Sanjo Finderle, Alojz Šmid

**Affiliations:** 1 Department of Gastroenterology, University Medical Centre Ljubljana, 1000 Ljubljana, Slovenia; peter.popovic@kclj.si (P.P.); rok.dezman@kclj.si (R.D.); jure.ursic@kclj.si (J.U.); martin.zaplotnik@kclj.si (M.Z.); miha.stabuc@kclj.si (M.Š.); sanjo.finderle@kclj.si (S.F.);; 2 Faculty of Medicine, University of Ljubljana, 1000 Ljubljana, Slovenia

**Keywords:** ischemic cholangiopathy, transarterial chemoembolization, hepatocellular carcinoma, biloma, bile duct dilatation

## Abstract

Background: Ischemic cholangiopathy (IC) is an under-recognized complication after transarterial chemoembolization (TACE) for hepatocellular carcinoma (HCC). Our goal was to estimate the incidence, timing, clinical presentation, and management of IC after drug-eluting bead TACE. Methods: Single-center retrospective cohort of consecutive drug-eluting bead TACE procedures (1 January 2020–31 December 2023) with imaging follow-up to 31 December 2024 was conducted. IC was defined radiologically as bile duct dilatation without mechanical obstruction, biloma formation, biliary strictures, or ischemic cholecystitis occurring after TACE. The primary outcome was the incidence of IC. Secondary outcomes included clinical presentation, need for hospitalization, and management. Results: Among 106 patients, 14 developed IC (13.2%). Imaging abnormalities were detected a mean of 3.1 months after TACE. Six patients (42.9%) were asymptomatic. Among symptomatic patients, the most common manifestations were abdominal pain (*n* = 6) and fever (*n* = 4). Five patients required hospitalization, including 2 with infected bilomas requiring antibiotics and/or drainage. Subsequent HCC therapy was feasible in most cases; no deaths were directly attributed to IC. Conclusions: IC after TACE is not rare and is frequently asymptomatic, often detected incidentally on routine CT follow-up. Systematic biochemical monitoring and selective MRCP could improve detection, particularly when TACE sessions are closely spaced.

## Simple Summary

Transarterial chemoembolization is an established treatment for hepatocellular carcinoma. It can sometimes affect nearby bile ducts by reducing their blood supply—a condition called ischemic cholangiopathy. In this retrospective single-center study, we examined the incidence of ischemic cholangiopathy, its clinical presentation, and its management. Among 106 patients who underwent the procedure, imaging signs of bile duct injury were found in about 13% of cases, usually appearing around three months after treatment. Nearly half of the affected patients had no symptoms, and the condition was often discovered incidentally during routine follow-up scans. When symptoms occurred, they typically included abdominal pain or fever, and only a few patients required hospitalization or specific treatment. Importantly, most patients were still able to continue therapy for liver cancer. These findings suggest that this complication may be more common than previously recognized and highlight the importance of careful imaging and clinical monitoring after treatment.

## 1. Introduction

Transarterial chemoembolization (TACE) is the gold standard treatment for hepatocellular carcinoma (HCC) at the intermediate stage, as defined by the Barcelona Clinic Liver Cancer (BCLC) staging system [1]. TACE exerts a dual antitumor effect through the locoregional delivery of a high dose of chemotherapy and by inducing tumor ischemia via embolic agents.

Generally, TACE is considered a safe and effective procedure with a relatively low incidence of complications. Reported complications include post-embolization syndrome, gastrointestinal bleeding, hepatic and renal function deterioration, liver infarction, iatrogenic dissection of the celiac artery, and, less commonly, pulmonary embolism [2]. One less frequently discussed but not uncommon complication is ischemic cholangiopathy (IC), resulting from disrupted blood flow through the peribiliary vascular plexus [3,4]. IC was first described in 1985 [5].

On imaging, IC can present itself as bile duct dilatation without a clear obstructive cause, bilomas, or strictures [3,6,7,8,9,10,11]. In case of clear ischemic etiology, ischemic cholecystitis is considered part of the IC spectrum [12,13]. However, there is inconsistency in the literature regarding the terminology and diagnostic criteria of these biliary changes, with some authors referring to them as ischemic cholangitis or biliary injuries. As a result, the true incidence, risk factors, clinical presentation, treatment strategies, and long-term outcomes of IC remain poorly defined.

The aim of this study was to evaluate the incidence, imaging features, clinical presentation, and management of IC after TACE and to describe our single-center experience with this complication.

## 2. Materials and Methods

### 2.1. Study Design and Patient Population

We conducted a retrospective observational study of patients hospitalized at the Department of Gastroenterology, University Medical Centre Ljubljana, who underwent TACE treatment of HCC between 1 January 2020 and 31 December 2023.

We included all consecutive patients who underwent their first TACE during this period, as well as those who had received TACE prior to 1 January 2020 but were still under treatment or follow-up within the study period. The decision on patient eligibility for TACE in the treatment of HCC was made by a multidisciplinary tumor board, taking into account the BCLC guidelines for hepatocellular carcinoma management.

Patients were excluded from further analysis if they had imaging features suggestive of IC before undergoing TACE or if no follow-up imaging was available after the procedure.

### 2.2. TACE Procedure

TACE procedures were performed by experienced interventional radiologists using drug-eluting bead TACE (DEB-TACE). Drug-eluting microspheres were loaded with idarubicin (10–15 mg) or doxorubicin (75–100 mg) and delivered via selective catheterization of tumor-feeding arteries. Embolization was performed using polyethylene glycol (LifePearl^®^ (Terumo Europe, Leuven, Belgium)) or sodium poly(methacrylate) microspheres (Tandem^®^ (Boston Scientific, Marlborough, MA, USA)) with a size of 100 μm. Whenever technically feasible, super-selective embolization of tumor-supplying branches was attempted to minimize non-target embolization.

### 2.3. Imaging Assessment

For all included patients, follow-up imaging studies performed until 31 December 2024 were reviewed. Imaging modalities included contrast-enhanced computed tomography (CT) and magnetic resonance imaging (MRI), which were performed as part of routine oncologic follow-up.

Radiological reports were reviewed for findings consistent with IC, which was defined as the presence of at least one radiological finding suggestive of ischemic biliary injury occurring after TACE in the absence of another identifiable cause. Radiological findings included bile duct dilatation without a clear obstructive cause, biloma formation, biliary strictures, and/or ischemic cholecystitis.

Imaging findings were based on routine clinical radiology reports, which were interpreted by experienced abdominal radiologists at our institution; no systematic re-review of imaging studies was performed specifically for the purposes of this study.

When present, the time interval between the TACE procedure and the first imaging finding suggestive of IC was recorded.

### 2.4. Clinical Data Collection

For patients with at least one imaging feature suggestive of IC, we conducted a review of medical records. This included outpatient clinic notes, hospital discharge letters, and other available medical documentation. We collected data on prior HCC treatments, relevant comorbidities, presenting symptoms, management strategies related to IC, and need for hospitalization and invasive interventions. Severity of complications was graded according to the Common Terminology Criteria for Adverse Events (CTCAE), version 5.0 [14]. Mild symptoms or asymptomatic findings not requiring intervention were classified as grade 1, events requiring medical therapy as grade 2, and events requiring hospitalization or invasive intervention as grade 3.

### 2.5. Study Outcomes

Our primary goal was to obtain data on the incidence of IC based on radiological criteria. Secondary outcomes were clinical presentation of IC, need for hospitalization, and specific treatment.

### 2.6. Ethical Considerations

The study was conducted in accordance with the Declaration of Helsinki (2013) [15]. Patient data were pseudonymized and accessible only to authorized study personnel and stored on secure institutional servers. Data processing was performed in compliance with the EU General Data Protection Regulation (GDPR). Informed consent was waived due to the retrospective nature of the study and the use of pseudonymized clinical data.

### 2.7. Statistical Analysis

Descriptive statistics were used to summarize patient characteristics and study outcomes. Continuous variables are presented as mean ± standard deviation (SD) or median with range, as appropriate. Categorical variables are reported as counts and percentages. Statistical analyses were performed using Microsoft Excel (Microsoft 365, Microsoft Corporation, Redmond, WA, USA). Given the descriptive nature of the study, which included summary statistics without inferential analysis, Excel was considered sufficient for data analysis. Due to the exploratory nature of the study and the relatively small number of IC cases, no formal risk factor analysis was performed.

## 3. Results

Between 1 January 2020, and 31 December 2023, a total of 119 patients who underwent at least one TACE procedure were hospitalized at the Department of Gastroenterology, University Medical Centre Ljubljana. Thirteen patients were excluded from further analysis: 5 had previously documented features of IC due to TACE performed prior to 2020, and 8 had no available follow-up imaging. Among these 8 patients, 5 died before follow-up imaging could be performed, 2 underwent liver transplantation shortly after TACE, and 1 had no available follow-up imaging for unknown reasons.

**Figure 1:**
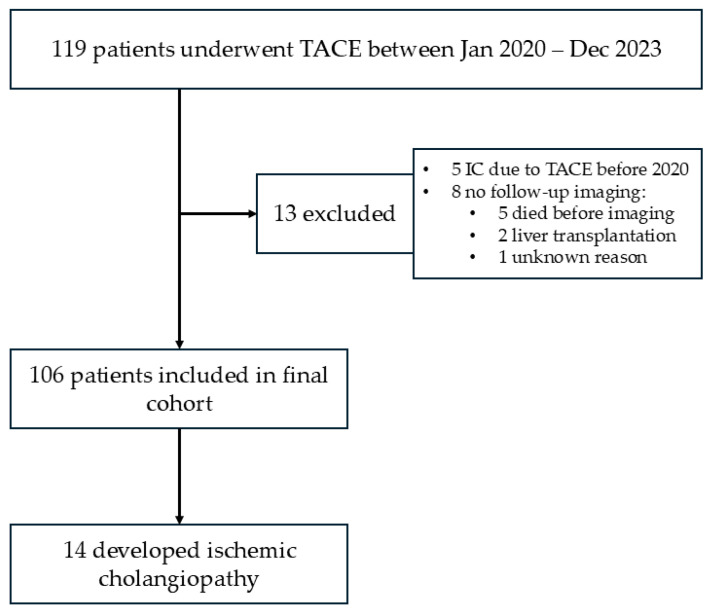
Patient selection.

The final study cohort consisted of 106 patients (95 males and 11 females). Imaging follow-up performed until 31 December 2024 identified radiological features consistent with IC in 14 patients (13.2%) (Figure 1).

### 3.1. Patient Characteristics

Among the 14 patients with IC, 13 (92.9%) were male and 1 (7.1%) was female. This sex distribution should be interpreted in the context of the overall study cohort, which showed a marked male predominance (95 males vs. 11 females), reflecting the baseline characteristics of patients undergoing TACE for hepatocellular carcinoma. Therefore, no conclusions regarding sex-specific susceptibility to IC can be drawn from these data.

The mean age at the time of the TACE procedure associated with IC development was 71.9 ± 6.7 years.

Liver cirrhosis was present in 11 patients (78.6%), most commonly alcoholic etiology (*n* = 9). Prior to the TACE procedure, 9 patients were classified as Child-Pugh class A and 2 as class B, while 3 patients had no evidence of liver cirrhosis. Eight patients had diabetes mellitus, and 7 patients had at least 3 comorbid conditions.

In 9 patients (64%), the largest HCC lesion treated during the TACE session associated with IC had a diameter < 50 mm. The median size of the treated lesion was 42.5 mm (range 15–70 mm).

Ten patients had undergone prior locoregional treatment for HCC, including TACE or microwave ablation, while 2 patients had a history of liver resection.

The patient characteristics are represented in Table 1.

### 3.2. TACE Sessions and Intervals

IC developed after the first TACE procedure in 4 (28.6%) patients. In 3 of these patients, TACE represented the first treatment for HCC, while 1 patient had previously undergone hepatectomy. Among these 3 patients for whom TACE was their first treatment, 1 patient had a non-selective embolization of the left hepatic artery during TACE.

The remaining 10 (71.4%) patients had at least one TACE before the TACE that resulted in IC. In 7 patients, IC developed after the second TACE, in 2 patients after the fourth TACE, and in 1 patient after the fifth TACE procedure.

The median interval between a prior TACE and the TACE causing IC was 29.5 days (mean 44.6 days, SD 41.9; range 27–163 days). In 9 of 10 patients (90%), two consecutive TACE procedures targeted the same lesion, with intervals ranging from 27 to 44 days (median 29 days, SD 5.2). One patient had a 163-day interval between procedures; however, in this case the second TACE targeted a different lesion than the first.

### 3.3. Diagnosis of Ischemic Cholangiopathy

Findings consistent with IC were first detected on CT imaging between 0.5 and 10 months after TACE (median 2.9 months, SD 2.4), representing the interval between the TACE procedure and the first CT scan demonstrating IC-related changes. In 2 patients, CT was performed due to clinical symptoms, whereas in the remaining 12 patients, IC was detected incidentally on routine follow-up imaging performed for HCC surveillance. No strictures of the bile ducts were reported on CT or MRI; however, none of the patients underwent MRCP.

**Figure 2:**
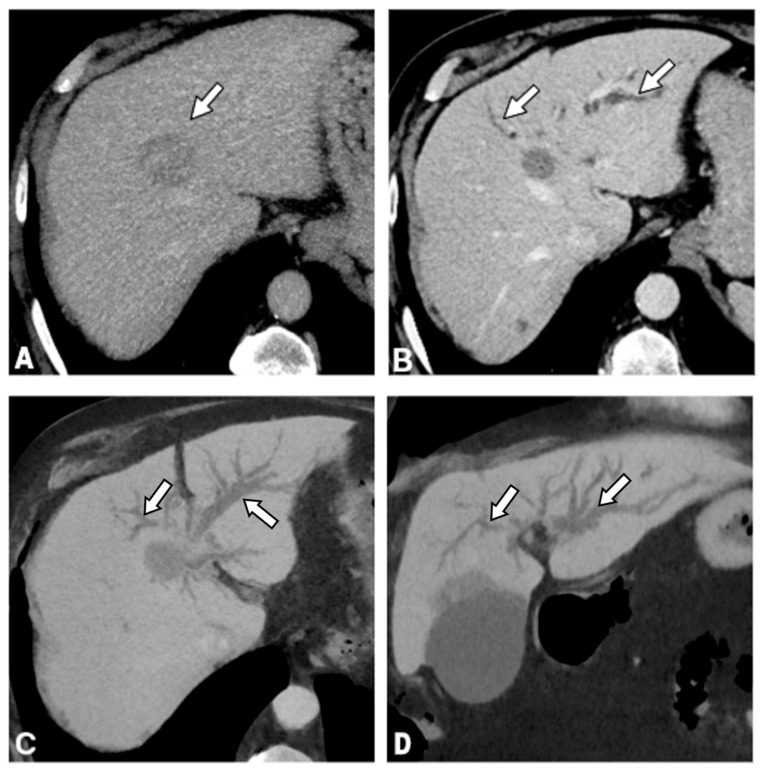
Bile duct dilatation after TACE in a patient, No. 2 from Table 1, with HCC. (**A**) Baseline contrast-enhanced CT shows HCC (arrow) in segment 4A adjacent to the left hepatic duct. (**B**) CT obtained 3 months after TACE demonstrates complete tumor necrosis, consistent with complete response, and newly developed diffuse bile duct dilatation (arrows) in the left hepatic lobe. (**C**,**D**) MinIP CT reconstructions better depict the dilated bile ducts (arrows) in the left hepatic lobe on axial (**C**) and coronal (**D**) images.

**Figure 3:**
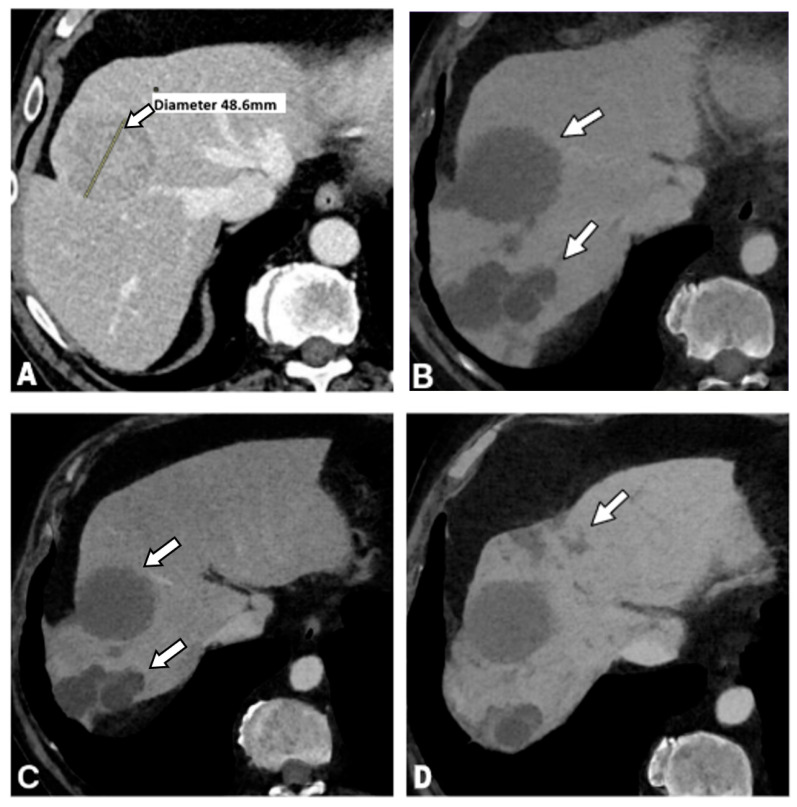
Evolution of ischemic cholangiopathy after TACE in patient No. 12 from Table 1 with HCC in a cirrhotic liver. (**A**) Baseline contrast-enhanced CT shows a 48 mm HCC (arrow) before treatment. (**B**) MinIP CT reconstruction obtained 3 months after TACE demonstrates two large bilomas (arrows) developing in the treated hepatic parenchyma. (**C**) At 12 months, CT shows slight regression of both bilomas (arrows). (**D**) On subsequent follow-up after 24 months and an additional TACE procedure, the bilomas are unchanged. while new focal bile duct dilatation (arrow) is visible.

The imaging manifestations of IC observed in the study cohort are summarized in Table 2. A sample of bile duct dilatation is shown on Figure 2 and of biloma on Figure 3.

### 3.4. Clinical Presentation and Management of Ischemic Cholangiopathy

Following the TACE procedures that led to the development of IC, 6 patients remained asymptomatic, while 8 patients developed at least one symptom.

The most frequently reported symptoms were abdominal pain (*n* = 6) and fever (*n* = 4). Loss of appetite occurred in 4 patients, 2 of whom also experienced weight loss. Nausea and vomiting were reported in 2 patients, and 2 patients described general malaise.

In 2 patients, symptoms persisted for longer than 60 days. According to CTCAE grading, 7 patients experienced grade 1 adverse events, 2 had grade 2 events, and 5 developed grade 3 complications requiring hospitalization or invasive treatment.

Five patients required hospitalization due to IC, with the length of stay ranging from 7 to 60 days (median 15 days). Two patients were hospitalized due to an infected biloma. One was treated conservatively with antibiotics because the infected biloma was too small for percutaneous drainage.

One patient was hospitalized for cholangitis, treated conservatively with antibiotics. Another was hospitalized due to unexplained elevation of inflammatory markers; retrospective review suggests the likely cause was cholangitis in the context of IC.

Two patients developed cholecystitis. One required hospitalization and underwent cholecystectomy shortly after TACE. Histopathology confirmed ischemic etiology due to embolic material found in the gallbladder wall. The second patient showed radiologic signs of cholecystitis on routine CT follow-up but was asymptomatic and managed conservatively without hospitalization. Both cases occurred after non-selective embolization of the right hepatic artery.

### 3.5. Further Treatment and Outcomes After Development of Ischemic Cholangiopathy

After the development of IC, 6 patients received at least one additional TACE procedure, 2 patients underwent microwave ablation (MWA), and 2 patients received systemic therapy due to HCC progression.

One patient who continued TACE developed an infected biloma requiring prolonged hospitalization, whereas no additional IC-related complications were observed in the remaining patients who received further locoregional therapies.

All patients who had been hospitalized due to IC later received further specific treatment for HCC. Dour patients experienced deterioration in performance status or liver cirrhosis decompensation, and best supportive care was initiated.

By 31 December 2024, three patients had died: 1 due to HCC progression, 1 from colon adenocarcinoma, and 1 from cardiovascular complications. None of the deaths were attributed to IC-related complications.

Overall, IC was most frequently detected incidentally on routine follow-up imaging and was asymptomatic in nearly half of affected patients.

## 4. Discussion

In this retrospective cohort of patients undergoing TACE for hepatocellular carcinoma, radiologic features of IC were identified in 13.2% of cases. IC was frequently asymptomatic and most commonly shown as isolated bile duct dilatation detected incidentally on routine follow-up imaging.

IC is a focal or extensive injury to the bile ducts caused by impaired perfusion of the peribiliary vascular plexus [3,5,6,7]. The bile ducts are supplied by a network of arterioles and capillaries forming a peribiliary vascular plexus, which originates from the hepatic arterial system [3,4]. Due to collateral circulation between arterioles and the arterioportal system intrahepatically and between hepatic and gastroduodenal arteries extrahepatically, ischemia of the bile ducts typically does not occur with proximal hepatic artery occlusion—except in the case of acute hepatic artery occlusion following liver transplantation—but rather due to obstruction of the small intrahepatic arteries [3,4,9].

The diagnosis of IC requires the presence of a clear trigger of ischemic biliary injury and evidence of bile duct injuries, for example, characteristic imaging findings or, rarely, histological features. TACE is among the most common causes of IC. Other known causes include liver transplantation, radiotherapy of the liver area, Rendu-Osler-Weber disease, systemic diseases with microvascular involvement (e.g., polyarteritis nodosa, Henoch-Schönlein purpura), AIDS-related cholangiopathy, and cholangiopathy in critically ill patients [3,9,14].

Imaging findings in IC include bile duct dilatation, biloma formation, and/or bile duct strictures [3,6,7,8,9,10,11]. These manifestations are likely related to the extent and rapidity of vascular compromise, as well as the time elapsed since the injury, and often coexist. Some authors suggest that initial epithelial desquamation and bile component accumulation lead to biliary cast formation, which, together with nonspecific reaction to the injury, presents on imaging as bile duct dilatation. More severe ischemia may cause focal transmural necrosis of bile ducts, leading to intrahepatic collections of necrotic material—i.e., bilomas [3,6,7,8,14,16]. Chronic or recurrent ischemia can result in bile duct strictures, which are indicative of severe injury and are associated with higher complication rates [3,6,7,17]. Between strictures, the bile duct can be dilated and therefore mimic the appearance of primary sclerosing cholangitis [3,9].

The most accurate imaging modality for diagnosing IC is MRCP [9,14]. CT is primarily effective for detecting dilated bile ducts and bilomas. However, CT is less sensitive for detecting biliary casts, stenosis, or subtle dilatation [3,6,10,18]. Despite its limitations, CT remains a valuable tool in diagnosing IC because it is used routinely in follow-up of response to HCC treatment and in urgent clinical scenarios.

Reported incidence rates of IC after TACE vary widely, ranging from approximately 1% to 22% [6,9,19,20,21,22,23]. This variability likely reflects differences in study populations, embolization techniques, and diagnostic criteria. In our cohort, IC was identified in 14 of 106 patients (13.2%), which is consistent with the upper range of previously reported incidence rates. Importantly, our definition of IC was based on radiological findings rather than clinically significant complications alone, which may partly explain the relatively higher incidence observed in our study.

According to some sources, bile duct dilatation is the most common imaging manifestation of IC, followed by bilomas, whereas biliary strictures are less frequent, reflecting the pathophysiological progression of ischemic biliary injury [6,10,20]. Our findings were generally consistent with these observations. In our cohort, bile duct dilatation was present in 12 patients and represented the sole manifestation in 9 cases. Bilomas were observed in 3 patients (isolated in 1). No biliary strictures were identified, which may relate to the predominance of CT in follow-up imaging.

Imaging abnormalities usually appear several months post-TACE—usually within 1 to 12 months, as also observed in our cohort [6,8,22,24].

A distinct form of ischemic injury is ischemic cholecystitis following TACE, usually caused by non-intentional embolization of the cystic artery. Acute cholecystitis typically develops within a few days of the procedure. However, there have been reports of many more patients remaining asymptomatic despite the presence of embolic material in the gallbladder wall and incidental radiological findings consistent with cholecystitis [5,9,12,13,24,25,26,27]. The reported incidence is 1–10% [9,12,27]. In our study, we documented ischemic cholecystitis in 2 patients (1.9%), with the need for cholecystectomy and consequently histological confirmation of ischemic etiology in 1 case.

Several prior studies have identified potential risk factors for IC after TACE. In patients with hepatic tumors, bile duct dilatation may preexist due to tumor compression, biliary stones, or prior surgical interventions. Such dilatation may promote chronic stasis and inflammation, increasing the risk of IC [22,28,29,30]. To minimize this confounding effect, patients with pre-existing biliary abnormalities were excluded from our analysis.

Some studies suggest that IC occurs more frequently in patients treated with DEB-TACE than with conventional TACE [6,16,18,23,29]. However, the role of chemotherapy dosage and embolic material volume in IC risk remains controversial [6,17,18,19,28,29,30], as does the impact of embolization selectivity [6,21,22,28,29,30].

Early reports suggested that the risk of IC increased with the cumulative number of TACE procedures, presumably due to repeated vascular injury and progressive arteriolar thrombosis [7,20,28,30]. However, more recent studies have shown that IC can occur even after a single TACE, with no consistent correlation between the number of TACE sessions and IC risk [16,17,18,19,22]. Guo et al. observed damage occurring after a mean of three TACE sessions and recommended close monitoring in patients undergoing more than two to three treatments [20]. In our cohort, IC developed after a variable number of TACE sessions, including after the second procedure in the majority of patients, which supports the notion that cumulative treatment number alone may not adequately explain the risk of ischemic biliary injury.

Shorter intervals (<90 days) between TACE sessions have also been proposed as a potential risk factor for IC [11,22,28]. In our study, 9 of 10 (90%) patients who had undergone multiple TACE sessions developed IC after a second procedure performed within 45 days of the previous treatment. Although the limited sample size precludes definitive conclusions, this observation is consistent with the hypothesis that closely spaced TACE procedures may increase the risk of ischemic injury, particularly when repeated embolization targets the same arterial territory.

There is stronger evidence linking IC to prior hepatectomy, particularly if TACE is performed within a short interval afterward [6,8,20,30]. Other locoregional therapies, such as microwave ablation, may also contribute to biliary injury [31] and could have influenced the observed findings in our cohort.

Some reports note a lower incidence of IC in tumors 5 cm or larger, likely due to their greater vascularization, which channels embolic agents into the tumor rather than surrounding tissue [6,22,32]. 9 patients (64%) in our cohort received a treatment for HCC smaller than 5 cm.

The role of liver cirrhosis in IC risk remains debated. Some studies suggest that cirrhosis may have a protective effect due to hypertrophy of the peribiliary vascular plexus and enhanced collateral circulation [16,21,22,28,32]. However, this effect has not been consistently demonstrated, and advanced cirrhosis may instead impair hepatic arterial perfusion, potentially increasing susceptibility to ischemic injury [8,18].

Consistent with previous reports [11,21,22,27,29,33], a substantial proportion of patients in our cohort were asymptomatic, with IC detected incidentally on routine imaging. When symptoms occurred, they were typically nonspecific, including abdominal pain, fever, malaise, or anorexia [3,9,19,34,35].

Laboratory abnormalities, particularly elevations in cholestatic enzymes such as alkaline phosphatase and gamma-glutamyl transferase, may provide an early indication of biliary injury [3,6,9,16,20,29]. However, laboratory monitoring in our cohort was not standardized, preventing reliable evaluation of biochemical predictors of IC.

Management of IC depends on clinical presentation. Asymptomatic patients may not require treatment, as bile duct dilatation and bilomas can regress spontaneously [3,9,22,29]. There is also no need for surgery in asymptomatic patients with radiological findings of post-TACE ischemic cholecystitis [25]. If infection is present, treatment is similar to that for abscesses, cholangitis, or strictures of other etiologies, i.e., antibiotics and percutaneous or endoscopic drainage [7,8,9,10,11,16,22,29]. Surgery may be necessary if drainage fails [6,7,8,16,22]. In our cohort, most patients were managed conservatively, although some required hospitalization or invasive treatment.

Long-term complications of IC after TACE may include portal vein branch stenosis, hepatic parenchymal atrophy, and intrahepatic lithiasis [6,10]. Patients with bile duct strictures, particularly at the hepatic hilum, tend to have worse outcomes due to the challenges of adequate treatment [6]. Estimated mortality from IC after TACE is 5–10% [6,8,19,22].

There is no established guidance for optimal HCC treatment after IC develops, nor is it clear whether further TACE is safe. Several studies opted not to continue TACE after the onset of IC [8,11,22]. Nakada et al. proposed that SIRT may be a safer alternative in these patients [8]. In our study, TACE was continued in 6 patients (42.9%). Only 1 patient developed additional complications, suggesting that further locoregional therapy may still be feasible in carefully selected cases. However, this approach should be considered with caution, and the decision to continue TACE after the development of IC should be individualized and made within a multidisciplinary team, considering the extent of biliary injury, liver function, and overall oncologic status.

Several limitations should be acknowledged. First, this study has a retrospective design and was conducted at a single center. Second, imaging assessment relied on routine clinical radiology reports interpreted by experienced abdominal radiologists, without systematic re-review of imaging studies for the purposes of this study, which may have resulted in an underestimation of subtle biliary abnormalities. Third, MRCP was not routinely performed, potentially limiting the detection of biliary strictures or casts. As a result, subtle biliary abnormalities such as strictures or biliary casts may have been underdetected. Fourth, laboratory follow-up was not standardized, which precluded analysis of biochemical predictors of IC. Finally, due to the limited number of IC cases and the descriptive design of the study, no formal analysis of potential risk factors was performed. A comparative analysis between patients with and without IC was beyond the scope of this study and would require a larger cohort to provide reliable results. Additionally, procedural parameters such as drug selection, dosage, microsphere size, and level of embolization were not systematically analyzed and were individualized based on tumor characteristics and vascular anatomy, which may have influenced the development of IC. Furthermore, some patients had undergone additional locoregional therapies, such as microwave ablation, which has been associated with biliary thermal injury, particularly in lesions adjacent to the biliary tree; therefore, the observed biliary abnormalities may be partially attributable to these treatments rather than TACE alone. These factors should be further evaluated in prospective studies specifically designed to assess treatment-related risk factors.

Despite these limitations, our study provides a detailed description of the imaging spectrum and clinical outcomes of IC after TACE in a real-world cohort and highlights the importance of recognizing this frequently underdiagnosed complication.

## 5. Conclusions

Ischemic cholangiopathy is a relatively common complication of TACE that can lead to significant clinical consequences. Due to limited awareness, this condition may be underdiagnosed and inadequately managed. Clinicians should maintain a high index of suspicion in patients presenting with nonspecific symptoms or unexplained elevations of cholestatic liver enzymes after TACE. Routine imaging follow-up should be carefully evaluated for subtle biliary changes, even in asymptomatic patients, and additional imaging such as MRCP may be considered when abnormalities are suspected.

Close clinical and biochemical monitoring are recommended, particularly in patients undergoing repeated or closely spaced TACE procedures. When feasible, efforts to minimize non-target embolization and to maintain adequate intervals between treatments may help reduce the risk of ischemic biliary injury. Decisions regarding continuation of TACE after the development of IC should be individualized and made within a multidisciplinary team.

## Figures and Tables

**Table 1:** Characteristics of 14 patients with ischemic cholangiopathy. CTCAE—common terminology criteria for adverse events; TACE—transarterial chemoembolization M—male; F—female; N/A—not applicable; BD—bile duct; HCC—hepatocellular carcinoma; SBRT—stereotactic beam radiotheraphy; MWA—microwave ablation; ECT—electrochemotheraphy; BSC—best supportive care; TKI—tyrosine kinase inhibitor.

Case No.	Age	Sex	Liver Cirrhosis			Prior HCC Treatment	Interval (Days) from Prior TACE	Type of IC	Clinical Presentation	Hospitalization (Length of Stay)	CTCAE Grade	IC Treatment	Further Treatment of HCC
			Present (Yes/No)	Etiology	Child-Pugh Class								
**1**	76	M	Yes	Alcohol	B	None	N/A	BD dilatation Biloma (infected)	Asymptomatic	Yes (60 days)	3	Antibiotics Drainage of infected biloma	TACE
**2**	78	M	Yes	Alcohol	A	TACE	28	BD dilatation	Fever + inapetence	No	1	No treatment	Without HCC progression
**3**	72	M	Yes	Alcohol + viral	A	TACE	34	Biloma (infected)	Abdominal pain	Yes (7 days)	3	Antibiotics	TACE
**4**	81	M	Yes	Alcohol	A	None	N/A	BD dilatation Cholecystitis	Fever + abdominal pain	Yes (16 days)	3	Antibiotics Cholecystectomy	TACE
**5**	70	M	Yes	Alcohol	A	TACE	29	BD dilatation	Asymptomatic	No	1	No treatment	SBRT, TACE
**6**	70	M	Yes	Alcohol	A	TACE	25	BD dilatation	Fever + abdominal pain + nausea and vomiting + malaise	Yes (12 days)	3	Antibiotics	TACE
**7**	73	M	No	N/A	N/A	Laparoscopic resection	N/A	BD dilatation	Inapetence + weight loss (<10% from the baseline) + malaise	No	1	No treatment	MWA
**8**	72	M	Yes	Alcohol	A	TACE	29	BD dilatation	Abdominal pain	No	2	Analgesic	TACE
**9**	61	M	Yes	Alcohol	B	None	N/A	BD dilatation	Asymptomatic	No	1	No treatment	BSC
**10**	68	F	No	N/A	N/A	Right hepatectomy, ECT, MWA, TACE	26	BD dilatation	Fever + abdominal pain + inapetence + nausea and vomiting	Yes (15 days)	3	Antibiotics	Immunotherapy
**11**	86	M	No	N/A	N/A	TACE	43	BD dilatation	Asymptomatic	No	1	No treatment	Without HCC progression
**12**	62	M	Yes	Viral	A	MWA, TACE	162	BD dilatation Biloma Cholecystitis	Abdominal pain + inapetence + weight loss	No	2	Analgesics	MWA
**13**	68	M	Yes	Alcohol	A	MWA, TACE	28	BD dilatation	Asymptomatic	No	1	No treatment	BSC
**14**	70	M	Yes	Alcohol	A	TACE	30	BD dilatation	Asymptomatic	No	1	No treatment	TKI

**Table 2:** Imaging manifestations of ischemic cholangiopathy observed in the study cohort (*n* = 14).

Imaging Finding	Number of Patients	Percentage
Bile duct dilatation	13	92.9%
Isolated bile duct dilatation	9	64.3%
Biloma	3	21.4%
Isolated biloma	1	7.1%
Infected biloma	2	14.3%
Biliary strictures	0	0%
Ischemic cholecystitis	2	14.3%

## Data Availability

The data presented in this study are not publicly available due to ethical restrictions. Data may be available from the corresponding author upon reasonable request and with permission from the relevant ethics committee.

## Abbreviations

The following abbreviations are used in this manuscript:

BCLC	Barcelona Clinic Liver Cancer
BD	Bile duct
BSC	Best supportive care
CT	Computed tomography
CTCAE	Common Terminology Criteria for Adverse Events
DEB-TACE	drug-eluting bead transarterial chemoembolization
ECT	Electrochemotheraphy
HCC	Hepatocellular carcinoma
IC	Ischemic cholangiopathy
MRI	Magnetic resonance imaging
MWA	Microwave ablation
N/A	Not applicable
SBRT	stereotactic beam radiotheraphy
SD	Standard deviation
TACE	Transarterial chemoembolization
TKI	Tyrosine kinase inhibitor
